# Groundwater Nitrogen Pollution and Assessment of Its Health Risks: A Case Study of a Typical Village in Rural-Urban Continuum, China

**DOI:** 10.1371/journal.pone.0033982

**Published:** 2012-04-13

**Authors:** Yang Gao, Guirui Yu, Chunyan Luo, Pei Zhou

**Affiliations:** 1 Key Laboratory of Ecosystem Network Observation and Modeling, Institute of Geographic Sciences and Natural Resources Research, Chinese Academy of Sciences, Beijing, China; 2 Institute of Agricultural Resources and Regional Planning, CAAS, Beijing, China; 3 School of Agriculture and Biology, Shanghai Jiaotong University, Shanghai, China; Pacific Climate Impacts Consortium, Canada

## Abstract

Protecting groundwater from nitrogen contamination is an important public-health concern and a major national environmental issue in China. In this study, we monitored water quality in 29 wells from 2009 to 2010 in a village in Shanghai city, whick belong to typical rural-urban continuum in China. The total N and NO_3_-N exhibited seasonal changes, and there were large fluctuations in NH_4_-N in residential areas, but without significant seasonal patterns. NO_2_-N in the water was not stable, but was present at high levels. Total N and NO_3_-N were significantly lower in residential areas than in agricultural areas. The groundwater quality in most wells belonged to Class III and IV in the Chinese water standard, which defines water that is unsuitable for human consumption. Our health risk assessments showed that NO_3_-N posed the greatest carcinogenic risk, with risk values ranging from 19×10^−6^ to 80×10^−6^, which accounted for more than 90% of the total risk in the study area.

## Introduction

Groundwater is the major water supply for drinking and for the domestic, industrial, and agricultural sectors in the Shanghai region of China. One serious problem that affects the quality of the region's groundwater is leaching of nutrients from the soil, which is especially evident in areas dominated by agriculture [1]–[2]. Nitrogen percolates easily into the groundwater through the soil along with rainwater recharge or irrigation water. As a result, the shallow aquifers are more likely than deeper ones to initially suffer from contamination problems [3]–[4]. The application of large amounts of nitrogen fertilizers in regions of intensive agriculture contributes to excessive nitrogen accumulation in soils and excessive leaching into groundwater bodies [5]–[7]. Extensive irrigation and use of nitrogen (N) fertilizers together result in low N-use efficiency and high N loss [8]. Several studies have also reported increasing incidence of nitrogen pollution and dramatic increases in the nitrogen concentration in the groundwater of regions where intensive farming is practiced [9]–[11].

Because contaminated groundwater resources are often located in the vicinity of wells for drinking water, it is essential to determine how management practices in the area surrounding these wells will affect groundwater nitrogen concentrations, and particularly nitrate nitrogen (NO_3_-N). Nitrate is formed from fertilizers, decaying plants, manure and other organic residues. It is found in the air, soil, water and food (particularly in vegetables) and is produced naturally within the human body. In many cases, groundwater nitrate concentrations are currently approaching or exceeding the recommended 11.3 mg NO_3_-N L^−1^ drinking water standard (e.g., [12]). Excess nitrates (levels >50 mg L^−1^; [13]) in the drinking water cause health risks such as conversion of hemoglobin to methemoglobin, which depletes oxygen levels in the blood. Forman et al. [14] reported additional consequences among people who consumed drinking water containing high levels of nitrates: enlargement of the thyroid gland, increased incidence of 15 types of cancer and two kinds of birth defects, and even hypertension. In addition, increasing rates of stomach cancer caused by increasing nitrate intake have been reported [15].

In Shanghai, nitrogen pollution has become an increasingly serious problem. Villages in the Shanghai city are the main areas for developing urban agriculture, which can provide the main source of vegetables and fruits for many residents. Due to extensive irrigation and fertilizer use, non-point source pollution is the dominant form, and the non-point source nitrogen loading has substantially affected groundwater nitrogen concentrations [16]. Poinke and Urban [17] showed that the average nitrogen concentration in rural groundwater was five to seven times higher than that in adjacent forest-covered areas. Where groundwater is the main source of drinking, domestic, and agricultural water, potentially significant health risks are associated with the consumption of nitrate-rich groundwater. For this reason, it is important to study the nitrogen pollution problem in rural-urban continuum near Shanghai to determine the impact on food safety and health of the residents. The aim of the present study was therefore to investigate seasonal changes in levels of nitrate and other forms of nitrogen, and based on this data, to assess the health risk for a typical village in Shanghai, thereby providing a scientific basis for controlling nitrogen pollution and protecting groundwater safety.

## Results

### Changes in different types of nitrogen in the groundwater of agricultural areas

**Figure 1:**
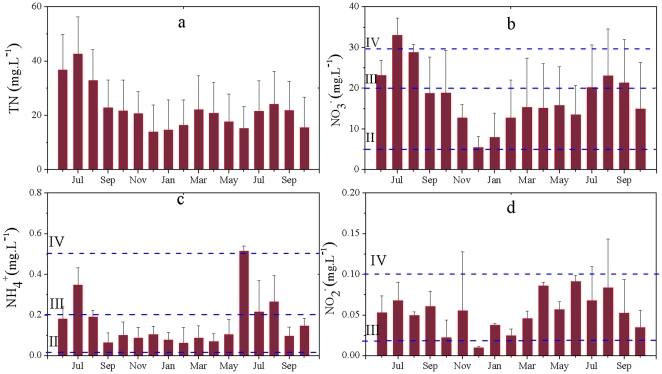
Seasonal changes in different types of nitrogen in the groundwater of agricultural areas of Xinchang village. Water quality grades are defined in Table 1. (a) total N; (b) nitrate nitrogen; (c) ammonia nitrogen; (d) nitrite nitrogen.

Figure 1 summarizes the results of the groundwater monitoring for the four types of nitrogen for wells in agricultural areas. The total N concentration was higher from June to August than during other months. The total N concentration exceeded 20 mg L^−1^ from June to August (Fig. 1a). From December to February, the total N concentration in groundwater reached its lowest value. Because NO_3_-N accounted for 60 to 80% of total N, the seasonal changes in NO_3_-N were similar to those for total N (Fig. 1b). According to the classification in Table 1, the groundwater quality for most wells from June to August was Class IV, with values ranging between 20 and 30 mg L^−1^, although some wells were rated Class V, with NO_3_-N exceeding 30 mg L^−1^; in the other months, the groundwater quality was rated Class III or worse.

**Table 1:** Classification standard for groundwater quality in China based on nitrogen levels. [18]

	Groundwater quality class				
	I	II	III	IV	V
Concentration (mg L^−1^)					
NH_4_-N	≤0.02	≤0.02	≤0.20	≤0.50	>0.50
NO_2_-N	≤0.001	≤0.010	≤0.020	≤0.100	>0.100
NO_3_-N	≤2	≤5	≤20	≤30	>30

Note: if NO_3_-N is class IV, it means that the concentration of NO_3_-N is between 20 to 30 mg L^−1^.

The degree of NH_4_-N pollution was an order of magnitude lower than the NO_3_-N pollution. The groundwater quality based on NH_4_-N level was rated as Class III in most months, except from June to July, when the quality degraded to Class IV (Fig. 1c). From September to May of the following year, the NH_4_-N concentration was relatively stable, decreasing to <0.2 mg L^−1^. The NO_2_-N concentration in groundwater was being in minimum from December to February in agricultural areas, but the change of NO_2_-N was mostly rated Class IV (Fig. 1d).

### Changes in different types of nitrogen in the groundwater of residential areas

**Figure 2:**
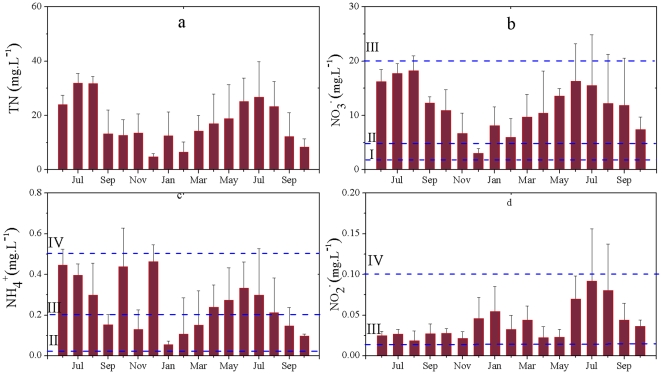
Seasonal changes in different types of nitrogen in the groundwater of residential areas of Xinchang village. Water quality grades are defined in Table 1. (a) total N; (b) nitrate nitrogen; (c) ammonia nitrogen; (d) nitrite nitrogen.

Total N concentrations in residential areas exhibited more gradual seasonal changes than in agricultural areas. In both the rainy season and the dry season, the total N concentration was significantly lower in residential areas than in agricultural areas (Fig. 2a). Although the NO_3_-N levels were lower in residential than agricultural areas, none of the wells met the criteria for Class I water and the wells only met the Class II standard in December (Fig. 2b). The residential NO_3_-N concentrations ranged from 5 to 20 mg L^−1^, and were therefore graded Class III. They showed a similar pattern of change to that in the agricultural areas.

The fluctuation in NH_4_-N concentrations in residential areas was large, but there was no significant seasonal change in NH_4_-N (Fig. 2c). The groundwater quality based on NH_4_-N levels ranged from Class IV to Class III, which was similar to the range in agricultural areas. The NO_2_-N concentrations were lower than those in agricultural areas, but the groundwater quality based on this pollutant was still graded as Class III or Class IV throughout the year, with a large increase in June to August 2010 but no substantive differences during the rest of the study period (Fig. 2d).

## Discussion

### Nitrogen pollution in the groundwater

In the study area, the highest concentration of NO_3_-N in the groundwater occurred from June to August, and NO_3_-N was the most significant nitrogen contaminant. The NO_3_-N concentrations were high from spring to summer and low from autumn to winter. During the jointing and booting stages of *Prunus persica* development, in March and April, most soil NO_3_-N would be taken up by the trees and by other vegetables. As the rainy season began after May in the Shanghai region, the total N and NO_3_-N levels in groundwater rapidly increased. From June to August, the NO_3_-N concentration in the groundwater of Xinchang was close to the limit prescribed by the World Health Organization [13]. Because NO_3_-N in solution is not adsorbed by soils, but NO_3_-N can easily be absorbed by some tropical soils and leach into the subsurface soil and groundwater [1], [5], [8].The changes in NH_4_-N levels were similar in agricultural and residential areas. However, the concentration of NH_4_-N was slightly lower in agricultural areas, indicating that the groundwater NH_4_-N was affected both by agricultural practices such as fertilization and by human habitation. The peak values of NH_4_-N content in the study area appeared from May to September, during the period of tree and vegetable growth and fertilization. Other inputs may come from agricultural production and domestic wastewater. Because nitrogenous fertilizers are applied to the soil, some of the NH_4_-N, which is a reactant for denitrification, can be transformed into NO_3_-N through nitrification, and some is lost as a result of denitrification to produce volatile nitrogen gas [11].

Although the concentrations of NO_2_-N were considerably lower than those of NO_3_-N in the groundwater, the impact of pollution by NO_2_-N was worse according to the Chinese groundwater quality criteria [18]; concentrations of NO_2_-N in the groundwater throughout the study area exceeded the Class III water standard, whereas NO_3_-N levels occasionally approached the Class II standard. NO_2_-N is not stable in water or soil, and can easily be transformed into NO_3_-N or into nitrogen gas through oxidation and denitrification. Therefore, the fluctuations of NO_2_-N concentrations were irregular and did not appear to be associated with seasonal changes as a result of impact factors such as changes in fertilization, rainfall, and temperature.

### Effects of rainfall and land use on nitrogen pollution

We found that land use patterns (here, residential vs. agricultural use) significantly affected NO_3_-N concentrations in the groundwater. Enhanced agricultural activity is often accompanied by increased incorporation of organic matter into the soil. Nitrogen compounds in the fertilizer and organic matter are transported into the groundwater by percolating water from rainfall or from irrigation [3]. Hence, the nitrogen concentrations are typically high in agricultural areas [19]–[21]. Another reason for this phenomenon may be that in agricultural areas, the aquifer is typically shallow, and because it is relatively close to the surface, it receives direct inputs of NO_3_-rich leachate from the agricultural soils. In residential areas, the nitrogen pollution was also serious, with levels close to those in agricultural areas. This can be explained by the high nitrogen content in groundwater around livestock and feedlot areas as well as near residences with septic tanks. Komar and Anderson [22] investigated the different nitrogen sources in a rural environment using nitrogen isotopes and obtained similar results to those in our study. Another reason for our observed results may be that the aquifers in the agricultural and residential areas are close to each other, so that leaching may transport pollutants between them; as a result, the magnitude of the difference in nitrogen contents in the groundwater would decrease.

**Figure 3:**
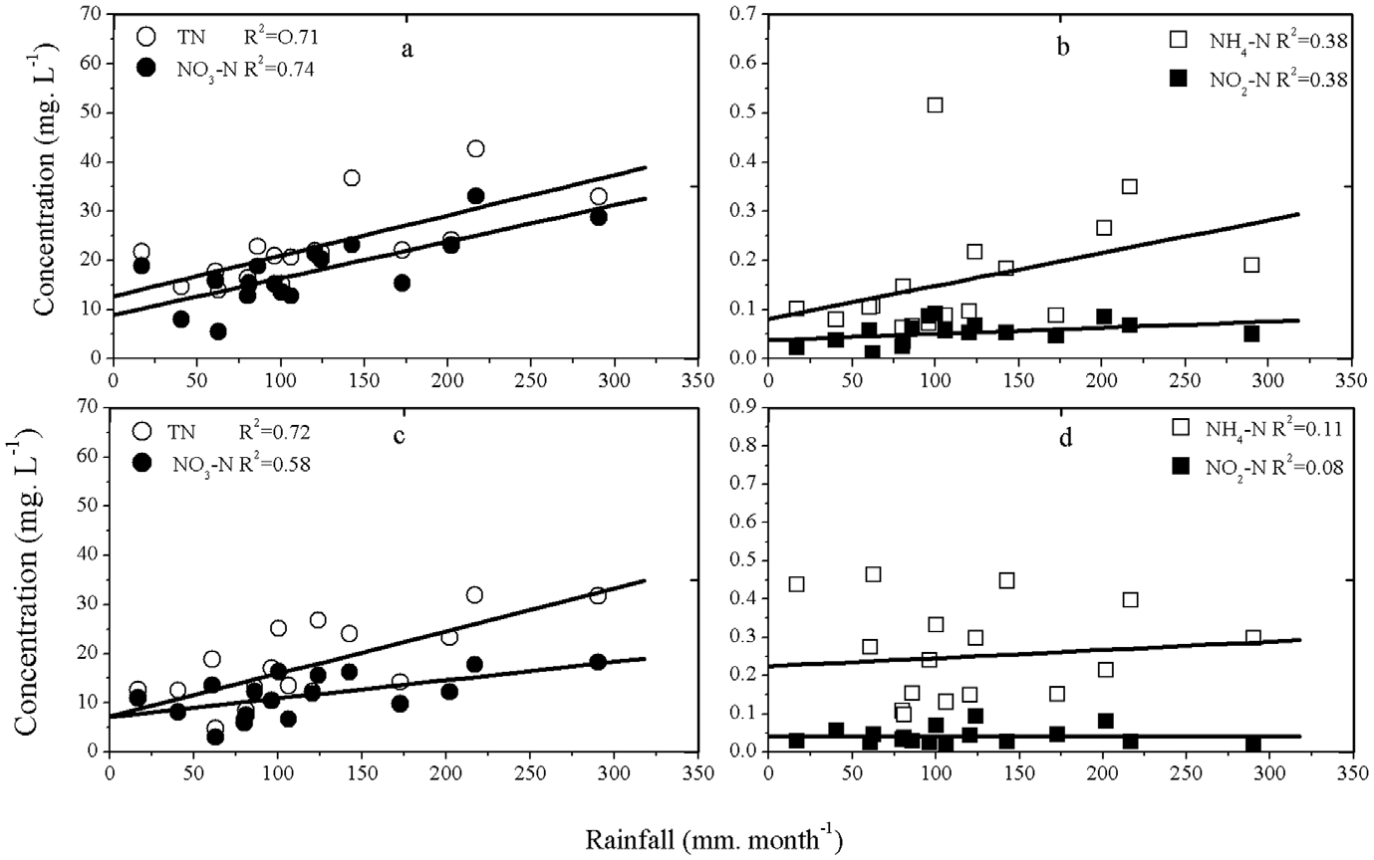
Relationships between the four types of nitrogen and rainfall. (a, b) agricultural areas; (c, d) residential areas.

The nitrogen concentrations in groundwater are affected by both rainfall and irrigation intensity [3], so we calculate the relationship between rainfall and the nitrogen concentration in groundwater (Fig. 3). The nitrogen concentrations in groundwater differed greatly between the rainy and dry seasons. The total N and NO_3_-N in the groundwater were significantly correlated with rainfall in both agricultural and residential areas, but the correlations between rainfall and NH_4_-N and NO_2_-N concentrations was much weaker but still significant. This can be explained by the fact that the abundant rainfall in the study area is the most important impact factor responsible for nitrogen transport through subsurface runoff into the groundwater, and by the fact that NO_3_-N accounted for 60 to 80% of total N. Soil nitrogen moves easily in water, especially during the first flush, when the runoff volume is high; internal and lateral solute movement in soils carried away nitrates even more intensively than surface runoff [23]. Zhu and Wen [24] showed that NH_4_-N is strongly absorbed by soil particles and is more resistant to being detached or dissolved and transported by runoff waters; this is because the ammonium ion has a positive charge and can therefore be adsorbed to cation-exchange sites on soil particles. NH_4_-N is easily oxidized or lost to denitrification, and NO_2_-N is not stable in water or soil. The other reason may be that soil pH in this area is 8.2, which easily affect NH_4_-N and NO_2_-N transformation via equilibrium. Therefore, NH_4_-N and NO_2_-N did not show a strong correlation with rainfall. The correlation between different types of nitrogen and rainfall was higher in agricultural areas than in residential areas. This is likely because in agricultural areas, groundwater nitrogen pollution was strongly influenced by agricultural activities such as irrigation and fertilization; human activities in residential area have less seasonal correlation than do activities in agricultural areas.

### Health risk assessment

Based on data for Shanghai from 2009 to 2010, the main parameters used in our health risk assessment had the following values: *IR* = 2 L d^−1^, *ED* = 30 years, *EF* = 365 d year^−1^, *BW* = 70 kg, *AT* = 70 years, *Asd* = 16 600 cm^2^, *FE* = 0.5 times d^−1^, *f* = 1, *k* = 1 cm h^−1^, *t* = 1 h, and *TE* = 0.4 h. The *Rfd* values for NO_3_-N, NO_2_-N, and NH_4_-N were 34, 1.6, and 0.1, respectively [25]. Potential noncarcinogenic risks for exposure to contaminants of potential concern were evaluated by comparison of the estimated contaminant intakes from each exposure route (oral, dermal, inhalation) with the *RfD*. The *HQ* assumes that there is a level of exposure (i.e., *RfD*) below which it is unlikely for even sensitive populations to experience adverse health effects. There may be a concern arising for the potential noncarcinogenic effects if the *HQ* exceeds 1×10^−6^ (unity).

**Figure 4:**
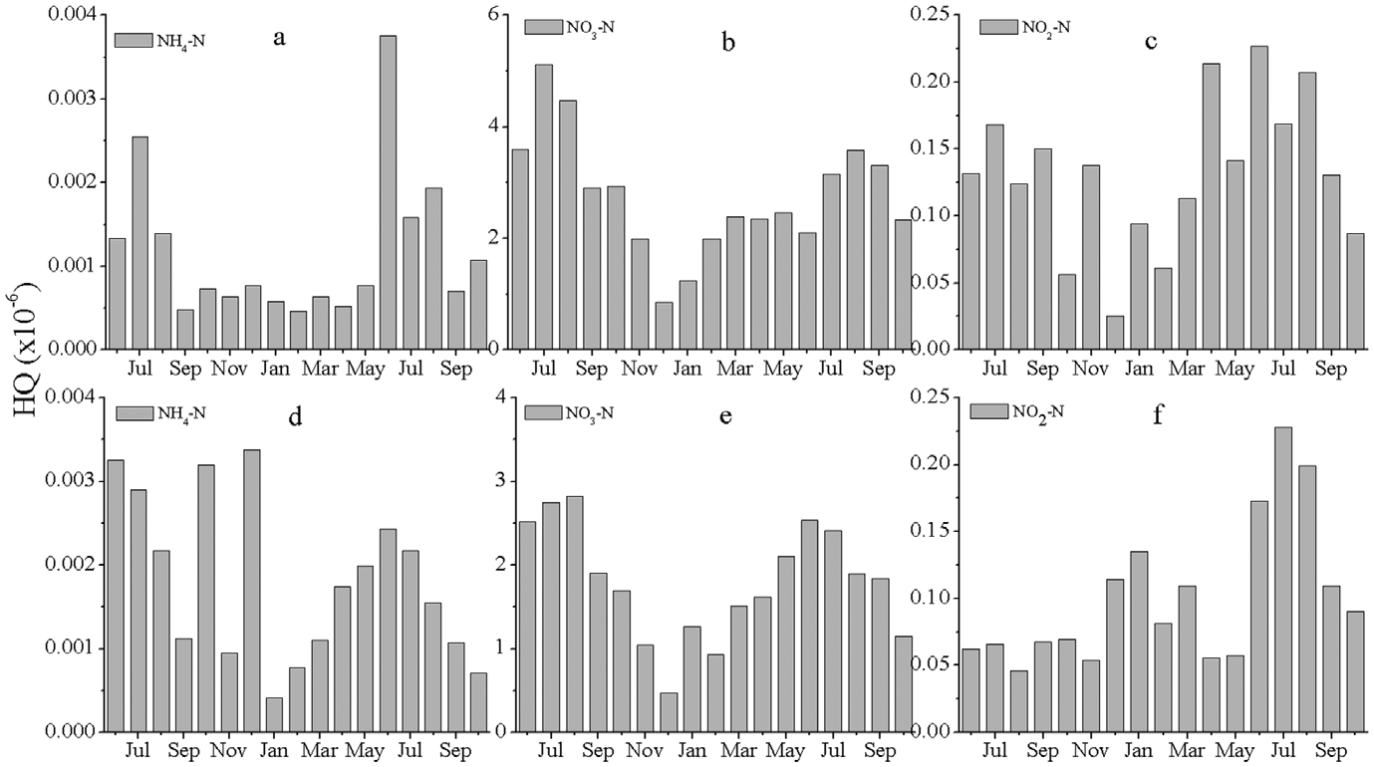
Noncarcinogenic dermal risk values for different types of nitrogen in the groundwater. (a, b, c) agricultural areas; (d, e, f) residential areas.

**Figure 5:**
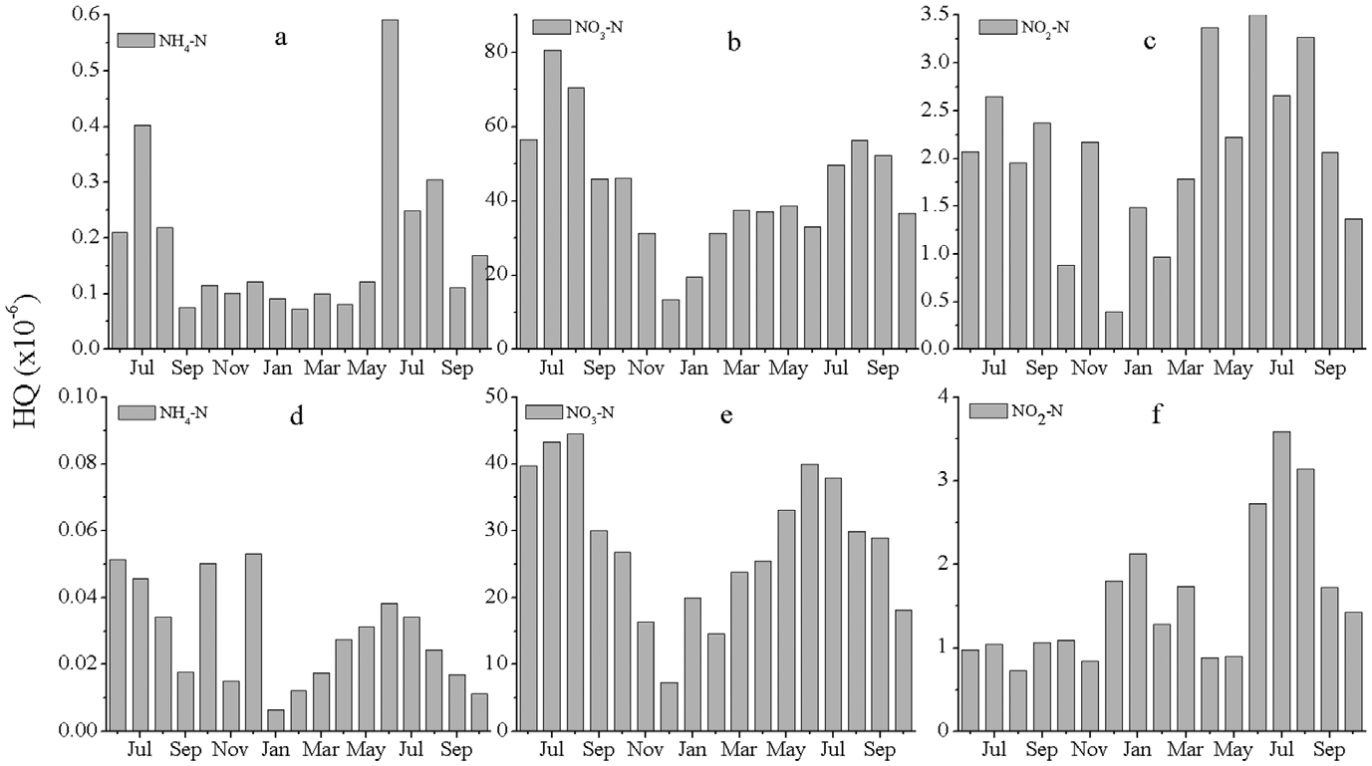
Noncarcinogenic oral risk values for different types of nitrogen in the groundwater. (a, b, c) agricultural areas; (d, e, f) residential areas.

Figures 4 and 5 present the noncarcinogenic risk values for dermal and oral exposures to different type of nitrogen, respectively. Drinking and contact were assumed to be the main exposure routes of humans to nitrogen pollution in our risk assessment; we did not include inhalation as a source of exposure, which may mean that our risk estimates slightly underestimate the actual risk. The relative research showed that the levels of noncarcinogenic oral risk, toxic risk (*HQ*), ranged from 0.02 to 0.12×10^−6^ [26].The noncarcinogenic dermal risks due to NO_3_-N and NO_2_-N showed seasonal changes, ranging from 0.8×10^−6^ to 3.5×10^−6^ (3.0 to 23.1 mg L^−1^)and from 0.05×10^−6^ to 0.22×10^−6^ (0.01 to 0.35 mg L^−1^), respectively. The *HQ* of the four types of nitrogen decreased in the following order: NO_3_-N>NO_2_-N>NH_4_-N. NH_4_-N represented the lowest noncarcinogenic dermal risk (Fig. 4). Noncarcinogenic dermal risk values were lower in residential areas than in agricultural areas.

The noncarcinogenic oral risk was two orders of magnitude higher than the noncarcinogenic dermal risk (Fig. 5). The levels of noncarcinogenic oral risk (*HQ*) ranged from 22×10^−6^ to 85×10^−6^. NO_3_-N posed the greatest risk, with HQ ranging from 19×10^−6^ to 80×10^−6^ (3.0 to 23.1 mg L^−1^); this accounted for more than 90% of the total risk in the study area. Therefore, NO_3_-N poses the greatest risk to human health. Daily intake of or contact with water by the local residents poses a potential health threat due to the cumulative impacts of long-term NO_3_-N exposure. The difference between the dermal and oral values indicates that ingestion of water is a more critical exposure route for NO_3_-N and NO_2_-N. The spatial variation in oral risk was similar to the spatial patterns of the dermal risk. The noncarcinogenic oral risk values were lower in residential areas than in agricultural areas. Local farmers irrigated their crops using groundwater, which would enhance the noncarcinogenic risk for residents in Shanghai. Government statistics reported one case of cancer for every 100 woman living in Shanghai, which was the highest cancer incidence in any Chinese city, and the number of cases of cancer was double time in 2010 than that in 1960s [27].

### Perspective

In China, there are many villages like Xinchang that are located in or near a big city, and which provide the main water resource and a supply of food and vegetables for city residents. Groundwater is the major source of water for drinking and for the domestic, industrial, and agricultural sectors in the Shanghai region. Therefore, protecting groundwater in this region has important implications for both food safety and human health. Because crops and animals take up nitrates from the soil and water, it will be important to quantify the nitrate contents of these foods and the quantities that are consumed in future research to determine how much this form of exposure increases the health risk to residents of Shanghai. In this study, our monitoring of the level of groundwater nitrogen pollution and our health risk assessment based on this data for Xinchang revealed that nitrogen pollution was a serious problem. Many wells exceeded the groundwater quality standard for human consumption for all forms of nitrogen, and particularly for nitrate, and higher levels of nitrogen contamination were significantly correlated with agricultural activity, human activity, and rainfall, especially in agricultural areas. The factors responsible for nitrogen pollution would be more complex in residential areas than in agricultural areas because of the greater diversity of activities. NO_3_-N was the main form of nitrogen pollution of the groundwater and poses the greatest risk to the health of local residents. Long-term drinking of groundwater and irrigation using groundwater therefore pose a significant health risk for Shanghai's residents. Therefore, it is urgent to devise policy guidelines for efficient management of both the surface water and groundwater resources in this region to enhance groundwater recharge and minimize the pollution levels in both types of water to permit their safe use.

## Materials and Methods

### Study area

**Figure 6:**
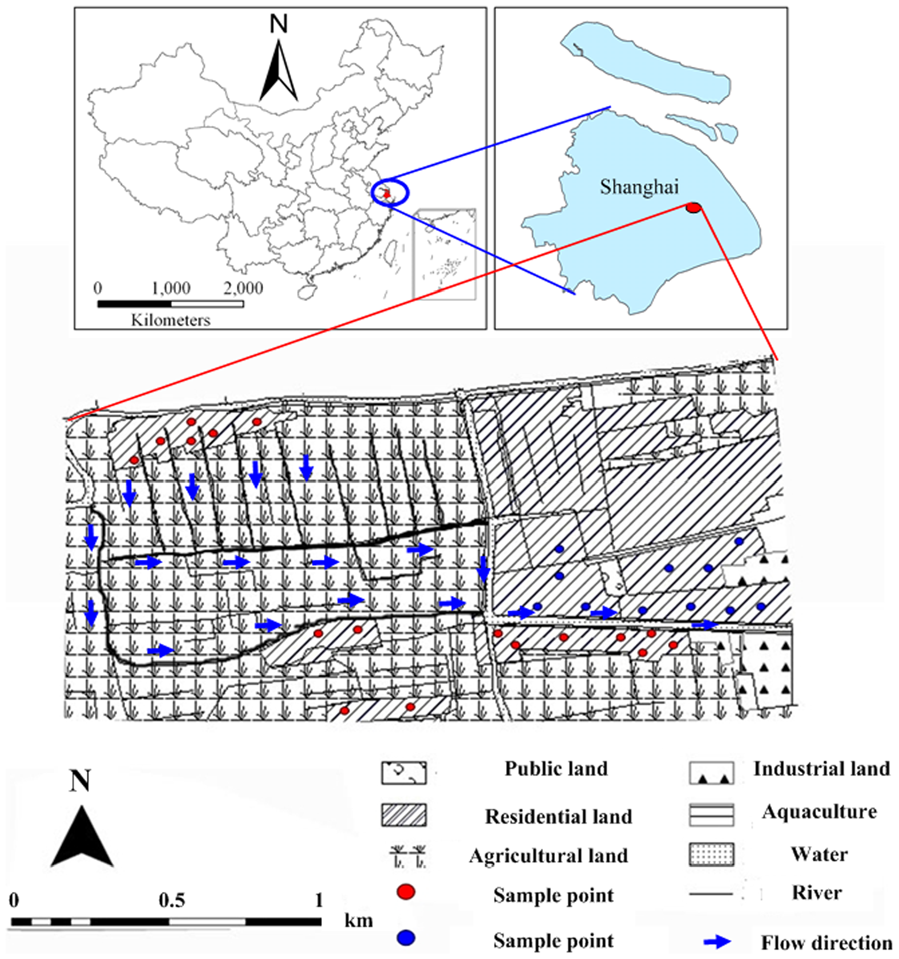
Location of the study area and sample points. 17 wells with red dot are in agricultural areas and 12 wells with blue dot are in residential areas.

The study area was Xinchang village in the Nanhui District of Shanghai (31°03′N, 121°39′E), which is located in a typical alluvial plain of the region. The village covers an area of about 3564 ha and has an altitude ranging from 2 to 3 m asl. The farming, livestock, and agriculture in this area are well developed. The main type of land use in Xinchang is planting of peach trees (*Prunus persica*) and vegetables, which account for more than 50% of the area. The residential population is around 1000 people (Fig. 6). The village's water system belongs to the Huangpu River watershed, where there are number of crossed rivers with abundant fresh water resources. The main streams of Huangpu River include Huixin, Dazhi and Fengxin River. Groundwater storage condition in this area depends on the pore water of the unconsolidated rock, so the groundwater complement source in Xinchang village is abundant. The climate is a subtropical marine monsoon climate, with average annual rainfall of 1175 mm, an average annual temperature of 16.7°C, and 1932 h of annual sunshine. The rainfall variation is large, and 70% of the rain falls from June to August.

The soil type is yellow clay in FAO Soil Classification [28]. The soil properties are a pH of 8.2±0.2, a bulk density of 1.2±0.2 g cm^−3^, a capillary porosity of 30.5±2.7%, a non-capillary porosity of 10.8±2.0%, a water content at field capacity of 13.6±1.0%, a total P of 0.8±0.2 g kg^−1^, an available P of 44.7±5.9 mg kg^−1^, an organic matter content of 20.4±0.7 g kg^−1^, and a total N of 106.6±4.4 mg kg^−1^ (n = 29). The local combined annual application of inorganic fertilizer equals 0.018 kg N m^−2^ plus 0.011 kg P_2_O_5_ m^−2^.

### Water samples

There are many wells in Xinchang, and the depth to groundwater level of this area generally ranges from 1.2 to 1.5 m. We set groundwater sample point according to the land use of the village and the principle of uniform distributed points through GPS positioning, and then record the latitude, longitude and water level information for different well. We sampled water from 17 wells (red dot) surrounded by agricultural areas and 12 wells (blue dot) in residential areas (Fig. 1). The water from wells with red dot is mainly used in daily need for local resident in agricultural areas and crop irrigation, whereas the water from well with blue dot mainly meet daily need from resident in residential areas. The domestic wastewater in residential area contains large amounts of organic nitrogen, wherein part of the domestic wastewater can directly leak into groundwater from sewer, and then cause groundwater nitrogen pollution [29]. Water samples were collected from June 2009 to October 2010 at 1-month intervals period. Groundwater samples (each 500 mL in size) were collected from pumps connected to the wells. Rainfall data were obtained from the local meteorological station in Nanhui District. All data is reported as means ± S.D.

### Analytical method

All water samples were passed through glass-fiber disks with a 0.70-mm pore size before analysis. To calculate total N, the water was digested in concentrated sulfuric acid using a CuSO_4_/Na_2_SO_4_ mixture as a catalyst, followed by distillation of the resulting NH_4_^+^ into dilute boric acid and titration against a standardized 0.0025 M H_2_SO_4_ solution, as described by Rayment and Higginson [30]. NO_3_-N was determined using an ultraviolet spectrometer [31], NO_2_-N was determined by means of diazo-coupling colorimetry [11], and NH_4_^+^-N was determined colorimetrically using the indophenol blue method [30].

### Groundwater quality and health risk assessment

We selected NO_3_-N, NO_2_-N, and NH_4_-N as the assessment index for groundwater nitrogen pollution. The national groundwater quality standard for nitrogen pollutants is presented in Table 1 [18]. Risk assessment is defined as the processes of estimating the probability of occurrence of an event and the probable magnitude of adverse health effects over a specified time period [32]. Human health risk assessment consists of four stages: (1) hazard identification, (2) toxicity (dose–response) assessment, (3) exposure assessment, and (4) risk characterization.

The estimated uptake of a potential toxin by the human body through contact with a contaminant is estimated using the chronic daily intake (*CDI*). The *CDI* value indicates the quantity of chemical substance ingested, inhaled, or absorbed through the skin per kilogram of body weight per day (mg kg^−1^ day^−1^). The formulas for calculating intake are as follows:

Ingestion:
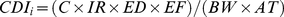
(1)Dermal contact:
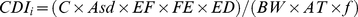
(2)

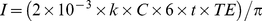
(3)where *i* represents a specific pollutant, *C* is that pollutant's concentration in water (mg L^−1^), *IR* is the drinking rate (L d^−1^), *ED* is the exposure duration (years), *EF* is the exposure frequency (d year^−1^), *BW* is the average body weight (kg), *AT* is the average lifespan (years), *Asd* is the human body's surface area (cm^2^), *FE* is the bathing frequency (number of times d^−1^), *f* is the intestinal absorption rate (unitless,  = 1), *I* is pollutant adsorption by the skin when bathing (mg cm^−2^ time^−1^), *k* is the adsorption parameter for the skin (cm h^−1^), *t* is the lag time (h), and *TE* is the bathing time (h).

### Noncarcinogenic risks

We separately characterized the risk for carcinogenic and noncarcinogenic effects, and have discussed the factors that may result in either overestimation or underestimation of the risks for the residents of Xinchang. Potential noncarcinogenic risks for exposure to contaminants were evaluated by comparison of the estimated contaminant intakes from each exposure route (oral and dermal) with the reference dose (*RfD*, (mg kg^−1^ day^−1^) to produce the hazard quotient (*HQ*, unitless), which is defined as follows [25]:
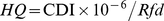
(4)where *HQ* is hazard quotient (unitless); *RfD* is reference dose (mg. kg^−1^ day^−1^).

### Carcinogenic risks

Carcinogenic risks were estimated as the incremental probability of an individual developing cancer over a lifetime as a result of exposure to a potential carcinogen. To do so, we used the following linear low-dose carcinogenic risk equation for each exposure route [25]:
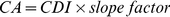
(5)where CA is the carcinogenic risk and “*slope factor*” is mg kg^−1^ day^−1^. Slope factor can be obtained from Risk Assessment Information System [33]. If a site has multiple carcinogenic contaminants, cancer risks for each carcinogen and each exposure route can be added (based on the assumption of additivity of effects) and compared with the accepted risk.
